# APOE Christchurch‐mimetic therapeutic antibody reduces APOE‐mediated toxicity and tau phosphorylation

**DOI:** 10.1002/alz.13436

**Published:** 2023-10-04

**Authors:** Claudia Marino, Paula Perez‐Corredor, Michael O'Hare, Annie Heuer, Natalia Chmielewska, Harper Gordon, Anita S. Chandrahas, Lucia Gonzalez‐Buendia, Santiago Delgado‐Tirado, Tri H. Doan, Timothy E. Vanderleest, Said Arevalo‐Alquichire, Robert A. Obar, Carolina Ortiz‐Cordero, Andres Villegas, Diego Sepulveda‐Falla, Leo A. Kim, Francisco Lopera, Robert Mahley, Yadong Huang, Yakeel T. Quiroz, Joseph F. Arboleda‐Velasquez

**Affiliations:** ^1^ Schepens Eye Research Institute of Mass Eye and Ear and Department of Ophthalmology at Harvard Medical School Boston Massachusetts USA; ^2^ Department of Cell Biology Harvard Medical School Boston Massachusetts USA; ^3^ Department of Chemistry Massachusetts Institute of Technology Cambridge Massachusetts USA; ^4^ Grupo de Neurociencias de Antioquia, Facultad de Medicina Universidad de Antioquia Medellín Colombia; ^5^ Molecular Neuropathology of Alzheimer's Disease Institute of Neuropathology University Medical Center Hamburg‐Eppendorf Hamburg Germany; ^6^ Gladstone Institute of Neurological Disease San Francisco California USA; ^7^ Gladstone Institute of Cardiovascular Disease San Francisco California USA; ^8^ Department of Pathology UCSF San Francisco California USA; ^9^ Department of Medicine UCSF San Francisco California USA; ^10^ Cardiovascular Research Institute UCSF San Francisco California USA; ^11^ Department of Neurology UCSF San Francisco California USA; ^12^ Department of Neurology Massachusetts General Hospital, Harvard Medical School Boston Massachusetts USA; ^13^ Department of Psychiatry Massachusetts General Hospital, Harvard Medical School Boston Massachusetts USA

**Keywords:** Alzheimer's disease, ApoE Christchurch, ApoE, Apolipoprotein E, drug development, heparan sulfate proteoglycan, heparin, HSPG, tau phosphorylation, tauopathy, therapeutic antibody

## Abstract

**INTRODUCTION:**

We discovered that the APOE3 Christchurch (APOE3Ch) variant may provide resistance to Alzheimer's disease (AD). This resistance may be due to reduced pathological interactions between ApoE3Ch and heparan sulfate proteoglycans (HSPGs).

**METHODS:**

We developed and characterized the binding, structure, and preclinical efficacy of novel antibodies targeting human ApoE‐HSPG interactions.

**RESULTS:**

We found that one of these antibodies, called 7C11, preferentially bound ApoE4, a major risk factor for sporadic AD, and disrupts heparin‐ApoE4 interactions. We also determined the crystal structure of a Fab fragment of 7C11 and used computer modeling to predict how it would bind to ApoE. When we tested 7C11 in mouse models, we found that it reduced recombinant ApoE‐induced tau pathology in the retina of MAPT*P301S mice and curbed pTau S396 phosphorylation in brains of systemically treated APOE4 knock‐in mice. Targeting ApoE‐HSPG interactions using 7C11 antibody may be a promising approach to developing new therapies for AD.

## 1 BACKGROUND

Alzheimer's disease (AD), the most common etiology of dementia, is characterized by amyloid beta (Aβ) plaques, tau fibrils, and neurodegeneration.^1,2^ The U.S. Food and Drug Administration (FDA) recently approved aducanumab for the treatment of AD based on evidence of reduced amyloid plaque burden from the brain.^3^, ^4^, ^5^ Additional trials were warranted by the FDA to demonstrate clinical benefits. Lecanemab, another amyloid targeting drug, recently showed a small but statistically significant delay in the progression of cognitive decline.^6^ Thus, previous work showed that it might be possible to modify the course of AD and clearly defines a path to clinical testing and approval for new and, hopefully, more effective therapeutics.

The link between specific Apolipoprotein E (*APOE*) variants and differential susceptibility to late‐onset AD starting after age 65 is one of the most studied and widely validated associations in human genetics.^7^, ^8^, ^9^ *APOE*ε3 occurs most frequently in 77% of the population and is by convention considered neutral regarding AD risk. *APOE*ε4 (Cys112 → Arg112) has a 15% frequency and is strongly associated with AD risk, explaining the genetic basis of 25% of cases. *APOE*ε2 (Arg158 → Cys158) occurs less frequently in only 8% of the population, adds a risk of hyperlipoproteinemia, and reduces AD risk.^10^ Our recent analysis of AD odds ratios in more than 5000 clinically and neuropathologically characterized cases along with control individuals confirmed that each additional copy of the *APOE4* allele was associated with a higher risk of AD, while *APOE* *2* homozygotes have a particularly low risk.^7^, ^8^, ^9^, ^11^

Familial AD (FAD) is transmitted as an autosomal dominant condition in subjects with mutations in one of three known genes: amyloid precursor protein (APP), presenilin 1 (*PSEN*1), and presenilin 2 (*PSEN*2). Mutations in these genes only explain around 5% to 10% of early‐onset AD starting before 65 years of age.^12^ We previously reported on the identification of the unique case of a Colombian woman who was genetically determined to develop AD dementia in her forties due to a *PSEN*1 E280A mutation but resisted the disease until her seventies.^13^ This subject had very high levels of amyloid in her brain, whereas tau pathology and neurodegeneration were lower than expected for her age.^14^ We discovered that this subject was homozygous for a rare variant in *APOE3* known as Christchurch (R136S, *APOE3Ch*), named after the city of New Zealand where it was discovered.^15^ The R136S Christchurch mutation is located in the domain involved in the interaction between ApoE and heparan sulfate proteoglycans (HSPG) and in the binding to the low‐density lipoprotein (LDL) and LDL receptor‐related protein (LRP) receptors.^13^, ^16^

Human and animal studies link ApoE and HSPGs to AD pathobiology,^8^, ^17^, ^18^, ^19^, ^20^ so ApoE‐HSPG interactions may be critical to AD progression.^3^, ^4^, ^21^ ApoE4, a major risk factor for sporadic AD, has a stronger binding to HSPGs, whereas the protective variants ApoE2, ApoE3Ch, and ApoE3 V236E (Jacksonville) bind less avidly to HSPGs.^22^ More recently, a major HSPG named Glypican‐4 was identified as a critical partner of ApoE4, mediating its toxicity and ability to propagate tau pathology in the brain.^21^ Genetic or competitive inhibition of glycosaminoglycans (GAGs), including HSPG, curbs spreading of neurotoxic tau seeds and modulates amyloid‐β clearance, aggregation, or toxicity in vitro and in AD mouse models.^18^, ^19^, ^20^, ^23^, ^24^ Therefore, modulating ApoE‐HSPG interactions might be critical in promoting resistance to AD‐related neurodegeneration.

Here, we developed and characterized ApoE‐Christchurch‐inspired antibodies (referred to as anti‐ApoE‐HSPG or anti‐ApoE‐GAG antibodies) capable of inhibiting the interaction between ApoE and HSPGs by using recombinant constructs of the HSPG domain of ApoE and ApoECh; we purified monoclonal antibodies and confirmed they competed for heparin (a type of GAG) binding and confirmed their ability to reduce ApoE‐derived cytotoxicity in vitro and tau phosphorylation in vivo. Our results led us to propose a novel antibody‐based platform for future clinical approaches targeting ApoE.

**RESEARCH IN CONTEXT**

1. **Systematic review**: We searched the Medline and PubMed databases for articles on therapies targeting ApoE for Alzheimer's disease (AD) to contextualize our findings. Other approaches have been developed and tested preclinically to reduce ApoE levels using biologics. However, we found no previous attempts to target ApoE using antibodies specific for the arginine residue at position 136 of *APOE* mutated in the *PSEN1* E280A mutation carrier (aka Christchurch case), which provides extreme protection against autosomal dominant AD (ADAD).
2. **Interpretation**: We designed and validated novel antibodies targeting ApoE at the site changed by the Christchurch variant. The uniqueness of our approach lies in the translation of genetic resistance to ADAD in individuals into an antibody‐based therapeutic approach.
3. **Future directions**: Our manuscript proposes a novel disease‐modifying target and therapeutic tools that will inform the future development of novel therapies against AD.

## 2 METHODS

### 2.1 Recombinant ApoE variants and anti‐ApoE‐GAGs antibodies

ApoE (KLH‐CTEELRVRLASHLRK‐CONH2) and ApoECh (KLH‐CTEELRVSLASHLRK‐CONH2) synthetic peptides, recombinant *Escherichia coli*‐derived His‐tagged full‐length (FL) human ApoE protein variants, antibodies from hybridoma, and purified monoclonal or human chimeric antibodies were produced as fee‐for‐service by Innovagen (Sweden). Recombinant Fab region including the complementarity‐determining regions (CDRs) of the 7C11.chIgG1 antibody was produced as fee‐for‐service by Creative BioStructure (Shirley, NY, USA). Mammalian‐derived (or HEK derived) ApoE variants were purchased from ACROBiosystems (Newark, DE, USA) (ApoE3, Catalog No. APE‐H5246; ApoE4, Catalog No. APE‐H52H9) or produced by the laboratory of the Kiessling Group at MIT. For the latter proteins, cDNA for APOE2, APOE3, and APOE4 with and without the Christchurch mutation were obtained as synthesized DNA gene fragments (gBlocks, Integrated DNA Technologies, Coralville, IA, USA), including the C‐terminal domain plus a His‐tag. gBlocks were Gibson ligated into a linearized modified pcDNA3 plasmid^25^ (primers: REV(5′‐GGTGAAGCTTAAGTTTAAACG‐3′) and FWD(5′‐TGAGGATCCACTAGTCCAGTG‐3′)). Correct insertion was verified by DNA sequencing (Quintara Biosciences, Cambridge, MA, USA). Each of the *APOE* isoforms was transiently expressed into Expi293F cells (Thermo Fisher Scientific, Waltham, MA, USA; Catalog No. A14528) using an ExpiFectamine293 Transfection Kit according to the manufacturer's instructions (Thermo Fisher Scientific). After 3 days, the conditioned expression medium was collected, centrifuged to remove cell debris, and steri‐filtered using a 0.2 μm polyethersulfone PES filter. Filtered supernatants were then loaded onto a HisTrap column (Cytiva) connected to a NGC^TM^ medium‐pressure liquid chromatography system (BioRad), washed with Ni‐NTA loading buffer (20 mM sodium phosphate, 500 mM sodium chloride, 20 mM imidazole, pH 7.4), and then eluted on a gradient to Ni‐NTA elution buffer (20 mM sodium phosphate, 500 mM sodium chloride, 500 mM imidazole, pH 7.4). For both cell culture and far‐western experiments, heparin was obtained from Galen Laboratory Supplies (North Haven, CT, USA; Catalog No. HO08). For in vivo treatments, mouse IgG1 isotype control (msIgG1) was purchased from Sigma‐Aldrich (St. Louis, MO, USA; Catalog No. M894).

### 2.2 ELISA

Clear polystyrene microplates (R&D Systems, Minneapolis, MN, USA; Catalog No. DY990) were coated with 0.0025 μg/μL of recombinant ApoE variants (ApoE2, ApoE3, ApoE4, ApoE3Ch; Innovagen) prepared in 2 mM CaCl_2_ 20 mM Tris Buffer saline (TBS‐C) and incubated for 16 h at 4°C. Subsequently, plates were washed two times with 2 mM CaCl_2_, 1X TBS‐T (TBSTC, 25 mM Tris HCl buffer 0.05% Tween 20; Thermo Fisher Scientific; Catalog No. 28360) and blocked for 1 h at room temperature (RT) with 3% BSA (bioWORLD, Dublin, OH, USA; Catalog No. 517986) in TBSTC. Upon washing the plates four times with TBSTC, serial dilutions of anti‐ApoE‐R136 antibodies (Innovagen) or anti‐mouse IgG1 isotype control (R&D Systems; Catalog No. MAB002) were prepared in blocking buffer and incubated for 2 h at RT or 16 h at 4°C. Plates were then washed four times and incubated for 45 min at RT with Horseradish Peroxidase (HRP)‐conjugated secondary antibody (1:10,000; Abcam, Cambridge, UK; Catalog No. ab97046) and 6 min with substrate reagent (R&D Systems; Catalog No. DY999) after washing the plates four times with TBSTC. To determine the competition of 7C11 antibody with VLDLr‐ApoE binding, 1 μg/mL of recombinant VLDLr (Creative BioStructure) was prepared in steri‐filtered PBS (R&D Systems; Catalog No. DY006) to coat ELISA plates by incubation for 18 h at 4°C under gentle shaking. Serial dilutions of ApoE3 (Acros), either alone or in the presence of 0.003 μg/μL of 7C11 mAb, were prepared in TBS‐C buffer and incubated for 2 h at RT. Samples were then incubated on the plate for 2 h at RT upon blocking the non‐specific binding using 3% BSA prepared in TBSTC buffer. Plates were then washed four times with TBSTC buffer prior to 2 h incubation at RT with anti‐His‐tag antibody (rb, 1:2,000, Abcam, Catalog No. AB9108). Subsequently, plates were washed four times prior to 45 min of incubation with secondary antibody (goat anti‐rabbit‐HRP antibody, 1:10,000, Abcam, Catalog No. AB6721). For both assays, the colorimetric reaction was detected spectroscopically as absorbance at 450 nm upon adding 2 N sulfuric acid (R&D Systems; Catalog No. DY994). Three independent experiments were conducted to ensure reproducibility. ELISA binding profiles were expressed as binding percentages normalized to the maximum absorbance.

### 2.3 Heparin affinity high‐performance liquid chromatography (HPLC)

We characterized heparin affinity chromatography using a Shimadzu SCL‐40 HPLC machine equipped with a POROS Heparin 50 μm Column (4.6 × 50 mm, 0.8 mL; Thermo Fisher Scientific) used as stationary phase and 20 mM Tris HCl pH 7.5 as mobile phase. To determine the affinity of the samples for heparin, we first loaded 50 μL of ApoE4 (50 μg/mL) either untreated or in the presence of 7C11 (95 μg/mL) using a flow rate of 0.3 mL/min kept constant for three column volumes (CVs). Subsequently, we increased the flow to 0.8 mL/min and tested heparin affinity via a multistep gradient of mobile phase up to 0.8 M NaCl in 20 mM Tris HCl pH 7.5, as follows: nine‐CV linear gradient from 0.1% to 20% 0.8 M NaCl in 20 mM Tris HCl, 12‐CV linear gradient from 20% to 70% 0.8 M NaCl in 20 mM Tris HCl, five‐CV isocratic elution with 99.9% 8 M NaCl in 20 mM Tris HCl, followed by equilibration of the column in 99.9% 20 mM Tris HCl for three CVs. Detection of the eluted fractions was conducted spectroscopically as fluorescence intensity upon exciting the samples at 227 nm and recoding the emission spectra at 350 nm. Analysis of retention times was obtained using both LabSolutions version 5.106 and GraphPad Prism 9 software. Data are representative of three independent experiments.

### 2.4 Biolayer interferometry (BLI)

The binding parameters of the 7C11 antibody variants were assessed using OCTET RED 96 at 25°C as a fee‐for‐service (Precision Antibody, Columbia, MD, USA). Anti‐mouse Fc (AMC), anti‐penta‐His (HS1K), and anti‐human IgG Fc (AHC) capture sensors were regenerated in 10 mM glycine buffer (pH 1.6) and pre‐equilibrated in PBS for 1 h. Upon determining the baseline in PBS, we performed the loading of msIgG1 7C11.mAb on AMC sensors and 7C11ch.IgG1 on AHC sensors for 480 s at 20 μg/mL. Subsequently, we recorded the baseline in PBS + 0.1% BSA for 120 s. Loading of antibodies (AMC or AHC sensors) was conducted to 480 s at a 20 μg/mL concentration. The association with the antigen was recorded for 420 s at 200 nM concentration and the dissociation for 800 s in PBS + 0.1% BSA. In parallel experiments, the same cycle was repeated using sensors that were not coated with antibodies at the loading steps as negative control. Data analysis was performed using Data Analysis software version 9.0 with parallel reference sensors.

### 2.5 Surface plasmon resonance (SPR) assay

Binding kinetics between the purified Fab‐7C11 protein and recombinant ApoE4 were quantified in PBS solution using a Biacore 3000 instrument (GE Healthcare) as a fee‐for‐service with Creative BioStructure and according to previously published procedures.^26^ Briefly, after chip activation, ligand printing, and chip blocking, the Fab was injected at various concentrations using the following settings: contact time = 120 s, dissociation contact time = 240 s, and flow rate = 30 μL/min. Data analysis was performed using BIAevaluation software. Kinetic parameters were monitored in real time to obtain on (ka) and off (kd) rates. The equilibrium constant (KD) was calculated from the observed ka and kd. Accuracy of the SPR analysis was determined via chi‐squared (χ2) analysis as described in the statistical analysis section.

### 2.6 Crystallography

We resolved the three‐dimensional crystal structure of purified 7C11.IgGFab protein as a fee‐for‐service with Creative BioStructure. Specifically, we conducted a high‐throughput crystallography screening with different kits using 27 mg/mL and 13 mg/mL peptide and the hanging drop method.^27^ Using a 96‐well sitting drop vapor diffusion plate, 50 μL of crystallization reagent from the deepwell block was dispensed into the reagent reservoirs of the crystallization plate. For the first screening trials, the crystal screen kits used for crystal screening were Index, Screen, Screen II, JCSG CORE I/II, PEG ION I/II, Natrix, Natrix II, Wizard I/II, and Wizard III/IV (Hampton Research or Qiagen). A hundred nanoliters of crystallization reagent from the crystallization plate reservoir were added to the sitting drop well and the equivalent volume of sample was transferred to the reagent drop in the sitting drop well. The crystallization plate was sealed using a clear sealing tape or film. Crystals were rapidly soaked in the reservoir solution supplemented with 20% glycerol as cryo‐protectant, mounted on loops, and flash‐cooled at 100 K in a nitrogen gas cryo‐stream. Crystal diffraction data were collected from a single crystal at Shanghai Synchrotron Radiation Facility (SSRF) BL17U beamline, China, with a wavelength of 0.9792 Å at 100 K. The diffraction data were processed and scaled with HKL3000. The structure was solved by the molecular replacement method^28^ with starting model PDB entry 5I1C, which has a 70% similarity with the 7C11.IgG1‐Fab sequence. The initial model was built using PHENIX.autobuild. Manual adjustment of the model was carried out using the COOT program, and the models were refined by PHENIX.refinement and Refmac5. The stereochemical quality of the structures was checked using PROCHECK. The structure was validated using the Protein Data Bank.^29^ All structure images were prepared using the PyMOL Molecular Graphics System (Schrödinger, LLC), UCSF Chimera, and BIOVIA Discovery Studio.

### 2.7 In silico analysis

Homology‐based modeling with the N‐terminal ApoE3 structure (PDB: 1NFN, ApoE aa sequence 23‐161, corresponding to ApoE aa 41‐181, or full length, UniProtKB: P02649) as reference was used to develop the ApoE3Ch model with Swiss‐Model in “user template mode” (https://swissmodel.expasy.org/). 7C11 and ApoE structures were uploaded to ClusPro 2.0 (https://cluspro.org) to model protein interactions using antibody mode and automatic mask of non‐CDR regions. For ApoE‐VLDr docking analysis, a homology‐based model was developed using SWISS‐MODEL in sequence mode. VLDLr repeats 5‐6 was chosen by the software as the template. Docking was done using ClusPro (https://cluspro.bu.edu/). Polar contacts between proteins were subsequently assessed in PyMoL using measurement mode.

### 2.8 Lactate dehydrogenase (LDH) assay

To test cytotoxicity changes with the different treatments, we used SH‐SY5Y (ATCC, Catalog No. CRL‐2266) cultured in DMEM/F12 (Gibco) enriched with 10% FBS (R&D Systems; Catalog No. S11150) at a density of 600,000 cells/mL 24 h prior to treatment. The following day, cells were washed one time with phenol‐free, serum‐free DMEM with 2 mM glutamax (Gibco) and treated with 1:2 serial dilutions of ApoE4, ApoE4Ch, vehicle (PBS), or a combination of 1 μM ApoE4 and 6.7 nM 7C11.chIgG1 or 13 nM heparin. Twenty‐four hours after treatment, controls (lysed cells and untreated cells) were generated, and 100 μL/well of LDH dye (Millipore, Catalog No. 4744926001) was incubated for 10 min at RT with culture media. The colorimetric reaction was stopped with 50 μL/well and cell toxicity measured as 490 nm absorbance. To determine the cytotoxicity percentage, we used the following equation: Cytotoxicity % = [X‐low control/(high control‐low control)] ×100. Further, to compare to ApoE4‐derived cytotoxicity, we normalized the cytotoxicity data to ApoE4, referred to as 100% cytotoxicity.

### 2.9 Far‐western blotting assay

Two micrograms of recombinant ApoE variants were prepared in non‐reducing Laemmli SDS sample buffer (Boston Bioproducts, Catalog No. BP‐110NR) and electrophoretically separated on a 4% to 20% pre‐cast gradient gel (Biorad) at 70 V for 10 min, followed by 90 V until complete separation. Proteins were transferred on nitrocellulose membranes (VWR; Catalog No. 27376‐991) using constant 400 mA for 1 h in 20% Methanol Tris‐Glycine buffer (Bio‐Rad). Membranes were blocked with 5% dry milk (M17200‐100.0, RPI) in TBST‐T for 1 h at RT with or without heparin (0.37 μM) and subsequently incubated with 7C11.mAb (0.018 μM) for 18 h at 4°C prior to detection using mouse IgG‐HRP‐conjugated secondary antibody (R&D Systems, Catalog No. HAF018), and subsequently 5 min incubation at RT with West Pico Super Signal PLUS Chemiluminescent Substrate (Thermo Fisher Scientific). Positive bands were acquired via chemiluminescence detection using a SyngeneG_Box Digital ECL detection system (Genesys software version 1.5.3.0). To qualitatively detect total levels of ApoE, membranes were incubated for 2 h at RT with goat anti‐ApoE (Millipore, Catalog No. AB947), washed three times for 10 min, and incubated with secondary antibody (IRDye, donkey anti‐goat 800 W, Licor, Catalog No. 926‐32214) for 1 h at RT prior to detection of ApoE‐positive bands using an infrared detection system (Odyssey, Licor; Image Studio Software version 2.1).

### 2.10 Animals and in vivo injections

We conducted in vivo intravitreal and intraperitoneal (ip) injections according to the Schepens Eye Research Institute Institutional Animal Care and Use Committee‐approved protocol and following institutional guidelines. Specifically, 41‐day‐old B6;C3‐Tg(Prnp‐MAPT*P301S)PS19Vle/J (Jackson Laboratory, Catalog No. 008169) male mice were injected with 2 μL of vehicle (PBS, pH 7.4), ApoE3 (50 μg/mL), or 7C11 (950 μg/mL). Three days after injection, animals were euthanized in a CO_2_ chamber. We enucleated the eyes and placed them in 4% paraformaldehyde (PFA, VWR) overnight at 4°C. The eyes were washed two times with PBS for 5 min. Retinas were dissected for subsequent staining procedures. Ip injections of either 7C11.mAb or mouse IgG1 were conducted on *APOE4* KI mice (B6.129P2‐Apoetm3(APOE*4)Mae N8, Taconic; ms.IgG1 group: three males, one female; 7C11.mAb group: three males, one female) at 16 to 18 months of age, which expresses human *APOE4*‐targeted gene replacement of the endogenous mouse *APOE* gene. Mice were treated with 20 mg/kg on day 1 and 3 consecutive days with 10 mg/kg. On day 5, mice were perfused with 4% PFA for immunohistochemical analyses of the harvested brains. To test the brain penetration of the antibody we also injected a separate cohort of *APOE4* KI mice with 50 mg/kg of Alexa‐647‐labeled 7C11.mab antibody or msIgG1 as control for 24 h prior to harvesting brain for further analyses.

### 2.11 Immunofluorescence staining of retina

Dissected retinas were blocked in dPBS (Gibco) supplemented with 1% donkey serum (Sigma‐Aldrich), 0.5% Triton X‐100 (Sigma‐Aldrich) for 48 h at 4°C under gentle shaking. Primary antibodies (Isolectin, 1:200, Thermo Fisher Scientific, Catalog No. I21411; anti‐phospho‐tau clone AT8, 1:500, Thermo Fisher Scientific, Catalog No. MN1020, and conjugated with Alexa‐594 conjugation kit, Abcam, Catalog No. AB269822) were prepared in dPBS with CaCl_2_ and MgCl_2_, 1% donkey serum, and 0.5% Triton X‐100, and retinas were incubated for 18 h at 4°C. Subsequently, retinas were washed with PBS every 10 min for 2 h and then incubated with DAPI (1:1,000 in PBS, Millipore, Catalog No. 10236276001) for 15 min, and subsequently washed every 15 min with PBS for 45 min in total. Prior microscopy acquisition, retinas were mounted using Vectashield fluoromount (Vector Laboratories, Catalog No. H‐1000‐10).

### 2.12 Immunofluorescence staining of murine brains

To test antibody penetration, APOE4 KI female mice were injected ip with vehicle (PBS), Alexa Fluor‐647‐labeled ms IgG1 (50 mg/kg), or Alexa Fluor‐647‐labeled ms.7C11‐Ab (50 mg/kg). Twenty‐four hours after injection, the mice were intracardially perfused with 4% PFA in PBS, and their brains were harvested and incubated in 4% PFA at 4°C for 24 h. Subsequently, their brains were washed in PBS and stained as described in the “Methods” section. Each brain was treated with a sucrose gradient (24 h 10% sucrose in PBS, 24 h 20% sucrose in 1X PBS, and 24 h 30% sucrose in PBS) for optimal cutting temperature (OCT) compound embedding, and sagittal sections were obtained at 8 μm thickness using cryostat while the other half was used for tissue clearing. OCT‐embedded sections were washed using PBS 1X and blocked with 3% BSA in 0.2% Triton X‐100. Sections were incubated with DAPI and subsequently imaged for quantification. Thirty images were taken at magnification ×40 per group. The fluorescence intensity of the 647‐nm channel was measured in MATLAB (version R2021A). Two independent experiments were conducted to determine the levels of antibody penetration. To determine changes in pTau levels upon 7C11.mAb treatment, females and males, both wild type (WT) and *APOE4* KI mice, were injected with mouse IgG1, 𝜿‐isotype control (Sigma‐Aldrich), or mouse 7C11.mAb antibody every 24 h for 4 consecutive days to reach a total dose of 50 mg/kg. Twenty‐four hours after the last injection, mice were intracardially perfused with 4% PFA 24 h after injection. A separate set of brains from WT and *APOE4* KI mice was collected as control groups for pTau. Brains were harvested and incubated with 4% PFA at 4°C for 24 h. Then brains were sectioned using a 1.0‐mm coronal stainless steel brain matrix. Brain sections corresponding to Bregma −1.75 to −2.75 were chosen to perform Clarity using a Binaree Tissue Clearing Kit (HRTC‐012) following the kit guidelines. After clearing, 1‐mm‐thick sections were incubated with anti‐pTau S396 (1:250, Thermo Fisher Scientific; Catalog No. 44‐752G). After 2 days of incubation with primary antibody, sections were washed and incubated with secondary antibody (1:500, Alexa Donkey anti‐rabbit) for 24 h. DAPI was added for 2 h, mounted, and subsequently imaged for quantification. Twenty randomized fields were captured at ×40 magnification per slide for each group. Every slide included the hippocampus, amygdala, cortex, and thalamus areas. For image analysis we wrote custom scripts in MATLAB (2021a) to quantify the number of cells that were positive for pTau S396 and the background‐subtracted fluorescence intensity of the cells. For both measurements we needed to segment the cells into images that contained non‐uniform illumination and noise. To segment the cells, we applied a difference of Gaussians filter (σ_1_ = 4 pixels, σ_2_ = 20 pixels) to the image and then used a hard threshold of 10 (au) for all images. The segmentation of the cells still contained some false positives that were smaller than the typical size of the cells, so we removed any connected components that were smaller than 70 pixels. The number of cells was taken to be the total number of connected components. To measure the fluorescence intensity, we used a Top‐hat filter (disk‐shaped structuring element with a radius of 20 pixels) to remove the background intensity from the raw image and then measured the average intensity over the mask of the cells. To obtain a mask of the signal from the cells, we used a difference of Gaussians filter (σ = 1 pixel, σ = 20 pixels) to improve contrast and then applied a threshold of 15 to obtain a mask of the cells. We performed a normality test prior to comparing the groups using the Kolmogorov‐Smirnov test. Statistical differences were determined using and the Kruskal‐Wallis one‐way ANOVA test for multiple comparisons and either Student's *t*‐test or Mann‐Whitney test when we compared two groups. Four animals per group were analyzed to determine statistical differences between treatment conditions.

### 2.13 Immunohistochemistry of *post mortem* human tissue

Middle temporal gyrus samples from *PSEN1* E280A female cases with different *APOE* genotypes (ε2ε3, ε3ε3, ε3ε4, ε4ε4, ε3ε3Ch, ε3Chε3Ch) were obtained from the NeuroBank of the University of Antioquia (*n* = 1). Brain tissue samples were formalin‐fixed, paraffin‐embedded (FFPE) and cut to 5 μm thickness. The slides were deparaffinized and rehydrated prior to staining. Citrate buffer and heat‐mediated antigen retrieval were used. Immunostaining was performed using an Epredia UltraVision Quanto Detection System HRP (Thermo Fisher Scientific TL060HL) according to the manufacturer's instructions. Briefly, slides were incubated in primary antibodies diluted in 1X TBS‐T buffer (Pierce) overnight at 4°C. We used anti‐Apolipoprotein E monoclonal antibody (clone E6D7, Abcam ab1907; 0.012 μg/μL) as the positive control and the new antibodies 1H4 (0.012 μg/μL), 7C11 (0.041 μg/μL), and 19G (0.072 μg/μL). Secondary‐only staining of some slides was used as negative control. All slides were scanned using a NanoZoomer SQ digital slide scanner (Hamamatsu) at the maximum magnification (×40). Images were analyzed using the NDP view 2 software (Hamamatsu).

### 2.14 Statistical analyses

Statistical comparisons for chromatography, in vitro and in vivo analyses were conducted using Graphpad Prism 9, and data were expressed as average ± SEM. Details regarding the replication for each experiment are reported in the figure legends. For the SPR data, we verified the accuracy of the results via χ^2^ analysis and compared experimental sensorgrams with the sensorgrams generated mathematically using BIAnalysis software. Values ranging from 1 to 2 were interpreted as significant (accurate) and those below 1 as highly significant (highly accurate). ELISA binding curves were analyzed by fitting a four‐parameter sigmoidal model or the area under the curve (AUC). Fitted models were assessed using Akaike's information criterion (AIC) to determine whether one or different curves were required to represent all the data sets. We compared AUCs using an unpaired *t*‐test or one‐way ANOVA together with a post hoc Tukey's test for multiple comparisons; *p* < .05 was reported as significant. Analyses of statistical differences in the histology data were performed using a one‐way ANOVA Kruskal‐Wallis test for multiple comparisons.

## 3 RESULTS

### 3.1 Specific antibodies bind to ApoE WT and ApoECh

We first synthesized peptides corresponding to the N‐terminal HSPG‐binding region (amino acids 114 to 144) of WT ApoE3 and ApoE3Ch variants. These peptides were used to induce an immune response in mice. We followed standard procedures to obtain hybridoma cell lines secreting novel anti‐ApoE antibodies with high specificity.

**FIGURE 1:**
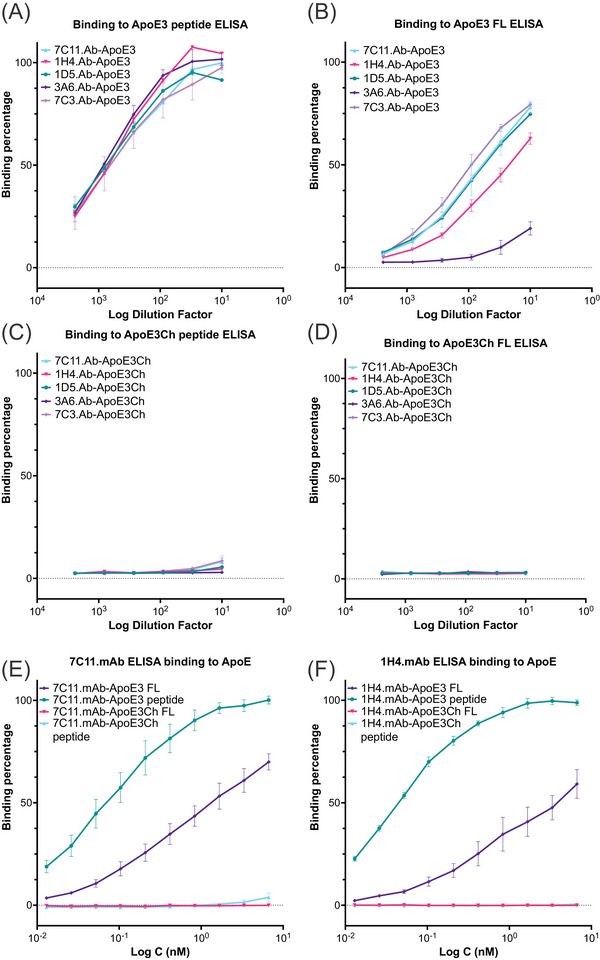
Anti‐ApoE3‐GAG antibody screening for top candidates. (A‐D) Screening of hybridoma cell supernatants via ELISA to test binding to peptides representing HSPG‐binding domains of ApoE3 (A), full‐length (FL) ApoE3 (B), ApoE3Ch peptide of ApoE3‐HSPG domain (C), and FL ApoE3Ch (D). Data show that all clones bind to HSPG region (ApoE peptide) with similar affinity (A) compared to binding between clones to the FL protein, in which clone 7C3.Ab (light purple) was the strongest, followed by 7C11.Ab (cyan), 1D6.Ab (green), 1H4.Ab (red), and 3A6.Ab (purple). Conversely, none of the antibodies tested showed binding for ApoE3Ch peptide (C) or FL ApoE3Ch (D), confirming the selectivity for the HSPG‐binding region of ApoE3. Binding expressed as averaged binding percentage normalized to the maximum intensity of absorbance as a function of logarithm of dilution factor ± SEM of three repeated measures. (E and F) Binding analysis via ELISA of purified monoclonal antibodies 7C11.mAb (E) and 1H4.mAb (F), using either FL protein (ApoE3 in purple and ApoE3Ch in red) or the amino acid sequence of the HSPG‐binding region (ie, peptide) of both ApoE3 (in green) and ApoE3Ch (in cyan). Data show differential binding for clones for either full‐length or small peptides of ApoE3 and ApoE3Ch. Binding analyses shown in panels (E) and (F) are expressed as averaged binding percentage normalized to the maximum intensity of absorbance over logarithmic nanomolar concentration (Log C) ± SEM of *n* = 3 repeated measurements.

We screened supernatants from the top five different hybridoma clones (7C11.Ab, 1H4.Ab, 1D5.Ab, 3A6.Ab, and 7C3.Ab clones) obtained from immunization with ApoE3 peptide and the top two hybridoma clones obtained upon ApoE3Ch peptide immunization (25F.Ab and 19G.Ab clones) via ELISA. As summarized in Figure 1, all clones bound to their corresponding ApoE3 peptide with similar binding profiles (Figure 1A). 7C3.Ab showed a strong binding affinity for full‐length ApoE3 (ApoE3 FL), followed by 7C11.Ab, 1D5.Ab, 1H4.Ab, and 3A6.Ab clones (Figure 1B). None of these antibodies showed detectable binding to ApoE3Ch peptide or to ApoE3Ch FL (Figure 1C,D), thereby validating a successful design of antibodies specific to the HSPG domain of WT ApoE.

We further tested purified mouse monoclonal antibodies (mAb) for the 7C11 and 1H4 clones chosen because of their strong reactivity to ApoE. As depicted in Figure 1E,F, ELISA binding profiles with either ApoE3 or ApoE3Ch confirmed the strong binding affinity of 7C11.mAb and 1H4.mAb for ApoE3. Upon comparing the AUC between 7C11.mAb and 1H4.mAb (Figure S1), we observed that 7C11.mAb bound to ApoE3 FL and had a higher AUC compared to 1H4.mAb (*p* = .0177, n1 = n2 = 21, q = 4.265, DF = 80, one‐way ANOVA), whereas the AUC for 1H4.mAb‐ApoE3 peptide binding was not significantly different compared to 7C11.mAb‐ApoE3 protein. None of the two antibodies were able to bind to ApoE3Ch peptide or FL, thereby confirming the selectivity for the WT ApoE3‐HSPG binding domain (Figure 1E,F). Conversely, 19G.Ab and 25F.Ab clones bound to their cognate ApoE3Ch peptide and ApoE3Ch FL protein, though they were not as selective and showed detectable cross‐reactivity for WT ApoE (Figure S2A‐D). This reduced selectivity may relate to the presence of different protein conformations in ApoE3Ch^13^ or because the amino acid sequence used to develop these clones shared a high similarity to ApoEWT. We observed a similar performance for the original 1343 antibody generated against the ApoE3Ch peptide in our original publication.^13^

We also quantified the binding of the purified 19G.mAb and 25F.mAb monoclonal antibodies to both full‐length ApoE3 and ApoE3Ch and short peptides representing the HSPG‐binding domain. We confirmed that both antibodies bound with stronger affinity the ApoE3Ch peptide, as expected (Figure S3).

We sequenced the CDRs for the monoclonal antibodies to identify specific amino acid sequences responsible for binding. CDRs for each clone are reported in Table 1. CDRs for all four antibodies have about twice as many of the acidic amino acids aspartate and glutamate (D, E; 10 to 12 amino acids) compared to the basic amino acids arginine, lysine, and histidine (R, K, and H; four to six amino acids), a feature that may be critical for interactions with the positively charged HSPG‐binding domain of ApoE.^30^ Light‐chain (VL) CDRs are almost identical among all four antibodies regardless of their binding for ApoE WT versus ApoE3Ch, whereas heavy‐chain (VH) CDRs are highly distinct, suggesting that some VH sequences may be critical for selectivity.

**TABLE 1:** CDR sequences of anti‐ApoE antibodies.

Clone	Chain type	CDR‐1	CDR‐2	CDR‐3
**7C11 VL**	Light	KASQSVDYDGDSYMN	AASNLES	QQSNEDPWT
**7C11 VH**	Heavy	RYTMS	KIRN**V**GGITYY**P** DTVKG	HYYGSEDYFDY
**1H4 VL**	Light	KASQSVDYDGDSYMN	AASNLES	QQSNEDPWT
**1H4 VH**	Heavy	**S**YTMS	KIRN**G**GGITYY**L** DTLKG	HYYGSEDYFDY
**19G10‐2 VL**	Light	KASQSVDYDGD **S**YMN	**A**ASNLES	QQSN**V** DPWT
**19G10‐2 VH**	Heavy	DYH **M** H	WIDPE **NGN**T**M**YDPKFQG	GTARASFDY
**25F1‐2 VL**	Light	KASQSVDYDGD **T**YMN	**T**ASNLES	QQSNEDPWT
**25F1‐2 VH**	Heavy	DYH **I** H	WIDPE **I** D KT**L**YDPKFQG	GTARASFDY

*Note*. For CDR‐1, CDR‐2 and CDR‐3 columns: bold font indicates amino acid changes. Green font indicates basic amino acids, and red means acidic.

### 3.2 Antibodies against the HSPG‐binding domain of ApoE preferentially bind specific ApoE isoforms

**FIGURE 2:**
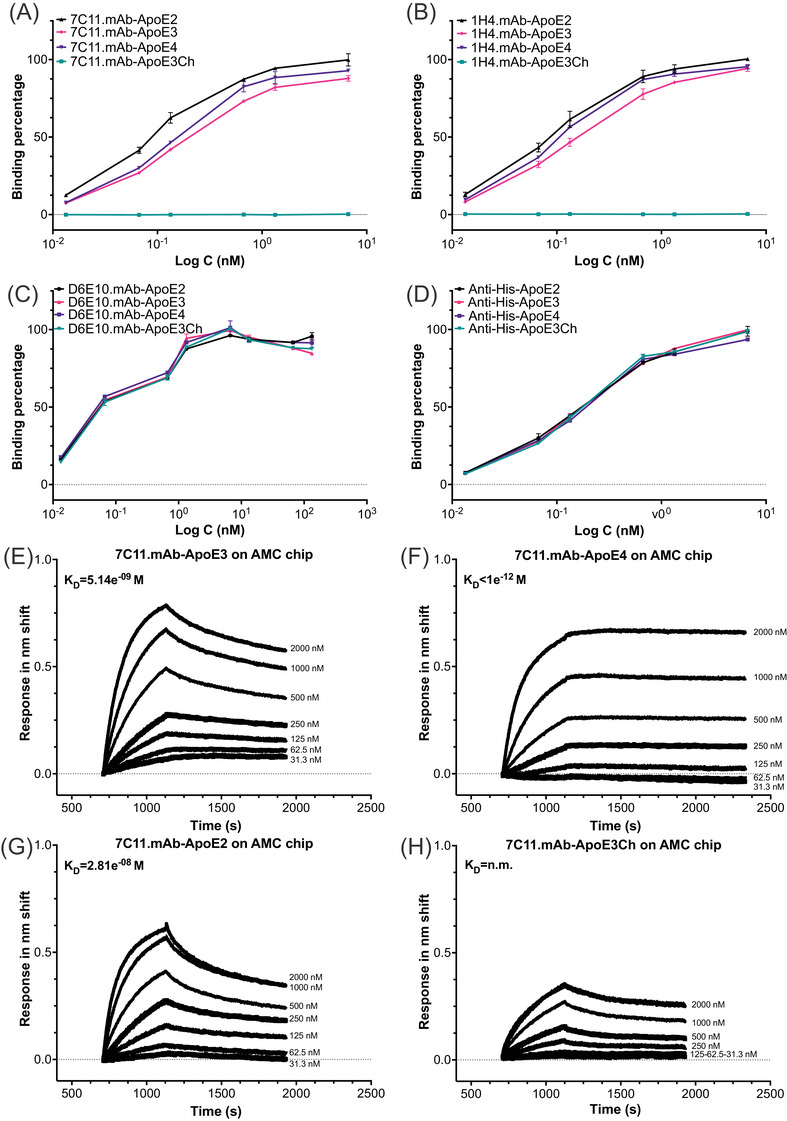
Assaying selectivity of anti‐ApoE‐GAG antibodies for ApoE variants of the novel ApoE antibodies. Titration of anti‐ApoE3 antibodies 7C11 (A), 1H4 (B), a commercially available anti‐ApoE antibody clone D6E10 (C), and a commercially available antibody anti‐His tag antibody (D). The assay was performed using a constant concentration of recombinant His‐tagged ApoE variants (ApoE2 in black; ApoE3Ch in cyan; ApoE3 in red; ApoE4 in purple). Data are expressed as binding percentage normalized to the maximum absorbance over logarithmic nM concentration and suggests that the 7C11 and 1H4 anti‐ApoE3‐HSPGs antibodies are more selective for ApoE2 and ApoE4 variants. (A‐D) All data are expressed as average binding percentage obtained from three independent experiments. (E‐H) Biolayer interferometry (BLI) binding analysis of ApoE3 (E), ApoE4 (F), ApoE2 (G), ApoE3Ch (H), and to immobilized 7C11 antibody to an AMC chip showing that 7C11 has the highest binding affinity for ApoE4, whereas it was not possible to measure binding affinity to ApoE3Ch. n.m. ≡ not measurable.

We examined the potency and selectivity of the 7C11.mAb, 1H4.mAb, and 19G.mAb monoclonal antibodies for different ApoE variants, including ApoE2, ApoE3, ApoE3Ch, and ApoE4. We tested the binding of serial dilutions of antibody concentrations ranging from 0.01 to 6.7 nM against a constant concentration (1.25 ng/μL) of ApoE2, ApoE3, ApoE4, and ApoE3Ch variants. As positive controls, we used the anti‐ApoE antibody clone D6E10, which targets the amino acid sequence 141 to 160 of ApoE (located outside the HSPG‐binding domain) and the anti‐His tag antibody, which are expected to recognize all our proteins (Figure 2A‐D). As negative control, we used the anti‐mouse IgG1 control antibody to confirm that the ApoE binding was specific (Figure S3). We observed that the 7C11.mAb and 1H4.mAb had similar binding profiles for their higher selectivity for ApoE2 and ApoE4 compared to ApoE3 and no binding to ApoE3Ch, when we expressed our data as binding percentage normalized to the maximum absorbance with the clone D6E10 antibody. Further, both 7C11.mAb and 1H4.mAb showed a similar potency for ApoE2 and ApoE4 in the low nanomolar range, as confirmed by the similar EC50 values (Table S1).

We further determined the binding constants of the anti‐ApoE antibodies using BLI (Figure 2E‐H), a technique more sensitive than ELISA. Specifically, we titrated ApoE2, ApoE3, ApoE4, and ApoE3Ch variants to determine the binding to 7C11.mAb coated onto the anti‐mouse Fc (AMC) chip. Our data showed that 7C11.mAb had a nanomolar affinity to ApoE3 (K_D_ = 5.14e^−9^ M) and a picomolar binding affinity for ApoE4 (K_D_ < e^−12^ M), which was significantly higher than the affinity for ApoE2 (K_D_ = 2.81e^−8^ M). This finding was surprising because the peptide used to raise 7C11 is identical for all ApoE isoforms. Conversely, the binding constant was not measurable (n.m.) for ApoE3Ch due to the lack of binding. Nevertheless, a minimal cross‐reactivity could not be excluded. We verified affinity under a different configuration by immobilizing ApoE variants on anti‐penta‐His chip (HS1K) and running titrating concentrations of 7C11.mAb. Under this configuration with the antibodies in solution and ApoE bound to the chip (Figure S3), 7C11.mAb had similar affinities among different isoforms (ApoE2 K_D_ = 7.41e^−9^ M, ApoE3 K_D_ = 1.68e^−8^ M, ApoE4 K_D_ = 6.07e^−9^ M).

Overall, we have produced a library of antibodies designed to target the HSPG‐binding domain of either ApoE3 or ApoE3Ch and characterized the binding profiles of the purified monoclonal antibodies 1H4.mAb, 7C11.mAb, 25F.mAb, and 19G.mAb, thereby allowing us to identify 7C11.mAb as a relevant candidate because of its high affinity for ApoE4, the strongest risk factor of late‐onset AD, possibly through the ability to interact with specific conformations of ApoE. 7C11's high affinity for ApoE3 is also clinically relevant as many patients with AD are APOE3 carriers. None of the newly designed antibodies showed any binding to mouse ApoE compared to anti‐His tag antibody used as positive control (Figure S4). This shows specificity for human ApoE while imposing some challenges for preclinical efficacy analyses in murine models.

### 3.3 7C11.mAb is a strong inhibitor of ApoE‐heparin binding in affinity chromatography

**FIGURE 3:**
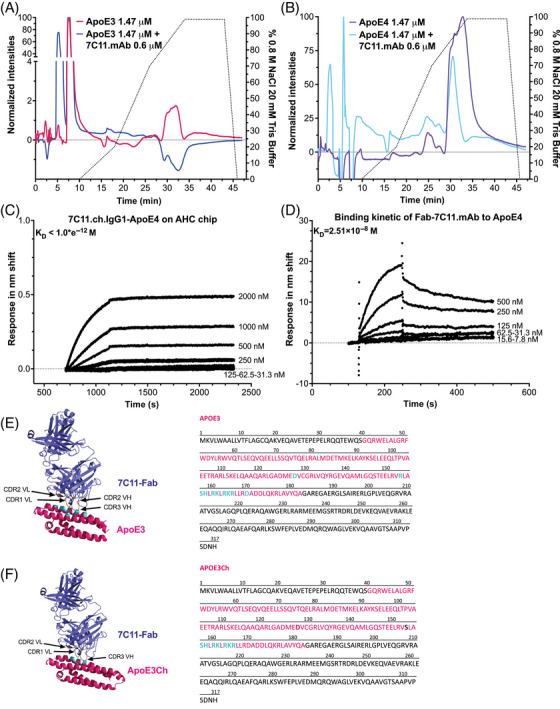
Affinity and in silico analysis of 7C11.mAb antibody. (A, B) Representative chromatograms of ApoE3 1.47 μM (magenta) and ApoE3 1.47 μM incubated with 7C11.mAb 0.6 μM (blue, A) or ApoE4 1.47 μM (purple) and ApoE4 1.47 μM incubated with 7C11.mAb 0.6 μM (light blue, B). Data show that 0.6 μM concentration of 7C11.mAb has a strong inhibitory effect on the heparin binding of ApoE3 and ApoE4, as confirmed by the shift of pmax at lower retention times and reduction of the peak intensity as compared to ApoE alone. All chromatograms are expressed as normalized intensities to the maximum intensity of emission over time in minutes (min) and are representative of *n* = 3 independent measurements. To compare the changes in ApoE, the 7C11.mAb chromatographic profile was subtracted. The percentage of 0.8 M NaCl over the salt gradient is represented by the dotted line. (C) BLI binding analysis of ApoE4 to immobilized 7C11.chIgG1 antibody to an anti‐human IgG Fc (AHC) chip showing that 7C11.chIgG1 binding affinity for ApoE4 with a K_D_ < 10^‐12^ M. (D) Binding analysis via surface plasmon resonance (SPR) of the Fab fragment of the 7C11.mAb antibody expressed as response difference over time of increasing concentrations of ApoE4 tested with immobilized Fab from antibody 7C11.mAb. (E, F) Representative in silico docking analysis of 7C11‐Fab antibody (in purple) to ApoE3 (E) or ApoE3Ch (F) N‐terminal region (in magenta). Polar contacts between the antibody and ApoE are represented in cyan. ApoE sequence for both ApoE3 and ApoE3Ch used for in silico analysis is reported next to the structures and highlighted in magenta; polar contacts within the sequence are highlighted in cyan.

We used heparin‐affinity chromatography to model the interaction between ApoE variants and HSPGs in a cell‐free system and measured the strength of interaction as a function of the concentration of NaCl required to elute either ApoE3 (Figure 3A, Figure S5A) or ApoE4 from the heparin column (Figure 3B, Figure S5B). Specifically, we injected samples in a 20 mM Tris HCl pH 7.5 mobile phase with no NaCl and maintained this eluting condition for three CVs to favor the interaction between ApoE and heparin. We progressively increased the concentration of NaCl to overcome ApoE‐heparin interactions for 21 CVs. Finally, we eluted an isocratic concentration of 0.8 M NaCl to favor the complete dissociation of the proteins from heparin for five CVs. Using this multistep elution gradient, we first determined heparin‐affinity chromatography profiles of ApoE3 and ApoE4. We observed that the retention time of the maximum normalized fluorescence peak (p_max_) for ApoE3 started at minute 29.8 to 30 (Figure 3A, Figure S5A), whereas for ApoE4 it was at minute 32.8 (Figure 3B, Figure S5B). We subsequently tested 7C11.mAb and confirmed the absence of binding between heparin and the antibody, as we observed all eluted peaks prior to the beginning of the NaCl gradient (Figure S5, cyan chromatogram).

We then tested both ApoE3 and ApoE4 binding to heparin in the presence of 7C11.mAb. We observed that 7C11.mAb effectively reduced the binding of ApoE4 to heparin using 50 μg/mL ApoE (1.47 μM) incubated with 95 μg/mL 7C11 (0.6 μM). Specifically, upon subtraction of the chromatographic peak of 7C11, we analyzed the chromatographic profiles of ApoE3‐7C11.mAb and ApoE4‐7C11.mAb. Our data revealed that in the presence of 7C11.mAb, the majority of ApoE3 and ApoE4 no longer bound to heparin, as confirmed by the multiple peaks eluted at minute 5.4 for ApoE3 (Figure 3A) and at minutes 2.8, 5.8, and 7.8 for ApoE4 (Figure 3B), prior to NaCl gradient elution, so 5.5 min became the new p_max_ for ApoE3 and the 5.8 min peak became the new p_max_ for ApoE4, respectively. Further, we no longer observed the peak at 30 min for ApoE3 and at 32.8 min for ApoE4, and the peak eluted at minute 30.7 was significantly reduced from 89.5% to 48.4% compared with the ApoE4 chromatogram. Overall, these data suggest that 7C11.mAb is a strong inhibitor of both ApoE3‐ and ApoE4‐heparin binding.

### 3.4 CDR grafting generated a chimeric mouse‐human IgG1 7C11 without loss of affinity to ApoE4

We generated a chimeric 7C11 anti‐ApoE antibody including mouse CDRs fused to human constant regions for IgG1. The chimeric 7C11 antibody bound ApoE4 with a similar affinity in the pico‐ to femtomolar range (K_D_ < 10^−12^ M, Figure 3C), thereby indicating that the CDRs identified in Table 1 are sufficient to support binding to ApoE. We sought to obtain the antibody crystal structure of 7C11 to better predict its interactions as a potential therapeutic. To this end, we synthesized the recombinant Fab fragment (7C11‐Fab), as this region would be easier to crystallize than the full‐length monoclonal antibody. As summarized in Figure 3D, we first validated the activity of the 7C11‐Fab peptide by quantifying the binding to ApoE4 via SPR. The binding analysis confirmed that the binding affinity of the 7C11‐Fab antibody was in the range of 10^−8^ M, which is lower than that of the full‐length antibody.

We used crystallography to determine the structure of the CDRs bound to the Fab protein fragment of the top candidate 7C11.chIgG1 antibody at a resolution of 1.76 to 1.70 Å. The structural details and refinement statistics of the crystallographic structure are reported in Table S2. We used the crystallographic structure of the 7C11‐Fab peptide to model in silico the interactions between the antibody and the R136‐interacting domain of both ApoE3 and ApoE3Ch. As reported in Figure 3E,F we confirmed that 7C11‐Fab makes many polar contacts (highlighted in cyan on both structure and amino acid sequence) with the R136‐binding site of ApoE3 and that position 136 (equivalent to codon 154 reported in the sequence displayed in figure 3D,E) is critical to favor the antibody‐protein interaction with ApoE3. Overall, our data confirmed that 7C11 interacts with ApoE3 at the R136‐binding domain, and this interaction is enhanced by the presence of an R at the amino acid 136 binding site: (1) polar bonds are predicted between R136 and the LE sequence from CDR2 VL and (2) an extended binding surface mediated by the R136 site may allow for antibody orientation of dipole moment involving positively charged amino acids in the HSPG‐binding domain and negatively charged amino acids outside the HSPG‐binding domain (D129, D170).^31^

### 3.5 Inhibiting ApoE‐HSPG binding reduced cytotoxicity in vitro and recombinant ApoE‐induced tau hyperphosphorylation in vivo

**FIGURE 4:**
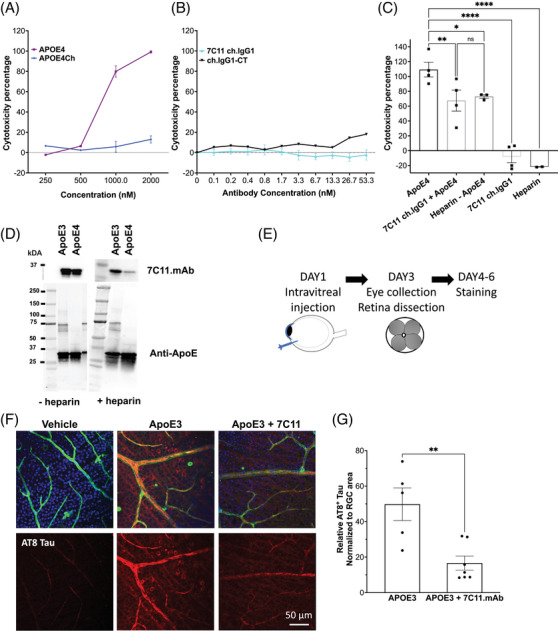
Introducing the Christchurch (Ch) mutation in ApoE4 or mimicking reduced GAG binding rescues cytotoxicity in vitro and tau pathology in vivo. (A) Cytotoxicity percentage of increasing concentrations of ApoE4 (purple) and ApoE4Ch (blue) used to treat for 24 h SH‐SY5Y cells showing the loss of toxicity via LDH assay in the presence of the Ch mutation. (B) Cytotoxicity assay of increasing doses of 7C11 human chimeric antibody (7C11.chIgG1) showing that it is not cytotoxic compared to IgG1 control. (C) Cytotoxicity assay comparing 1 μM ApoE4‐treated cells to 1 μM ApoE4 co‐administered with either 7C11 human chimeric antibody (7C11.chIgG1 6.7 nM) or heparin 13 nM showing that ApoE4‐derived cytotoxicity is significantly reduced in vitro (*p = .0147, unpaired two‐tailed *t*‐test, *n* = 4 biological replicates). Neither heparin nor 7C11 were cytotoxic. Data were normalized by considering ApoE4 cytotoxicity as 100%. (D) Representative far‐western blotting of ApoE3 and ApoE4 showing that in the absence of heparin (left blots), 7C11 binds more to ApoE compared to blots probed in the presence of heparin (right blots). Total levels of ApoE were not affected when membranes were probed with anti‐ApoE antibody (botton blots). (E) Pictographic representation of experimental design of in vivo intravitreal injections of ApoE variants or vehicle or ApoE3 + 7C11.mAb performed on day 1, euthanasia and retina dissection from the collected eyes on day 3, and staining on day 4 after injection. (F) Representative immunofluorescence staining of dissected retina injected intravitreally with 2 μL of vehicle (PBS), ApoE3 1.47 μM, or ApoE3 1.47 μM + 7C11.mAb 6 μM, showing reduction of retinal damage and inflammation, as shown by increased levels of isolectin staining (green) and AT8‐tau staining (in red) in the presence of 7C11.mAb antibody. For all images, the top panel is a composite of staining obtained with DAPI to detect nuclei (blue) and AT8‐tau to detect hyperphosphorylated tau accumulation (AT8, red), isolectin B4 (green) to detect vasculature. The red channel is also displayed in the bottom panel. Scale bar = 50 μm. (G) Quantification of the relative AT8‐positive tau levels normalized to retinal ganglion cell (RGC) area in ApoE3 and ApoE3 + 7C11.mAb‐treated retinas showing a significantly reduced AT8‐tau staining in the presence of 7C11.mAb compared to ApoE3 alone (**p = .0042, unpaired two‐tailed *t*‐test, *n* = 3, 4 biological replicates).

To examine the in vitro efficacy of our antibodies, we sought to optimize an ApoE4 toxicity assay. As a positive control, we tested whether introducing the Christchurch mutation into ApoE4 (ApoE4Ch) would reduce cytotoxicity in vitro. As shown in Figure 4A, we used a LDH assay to measure changes in the cytotoxicity of SH‐SY5Y neuroblastoma cells treated for 24 h with increasing concentrations of ApoE4 and ApoE4Ch, respectively. Our data strongly suggest that introducing the Christchurch mutation on ApoE4 abolishes cytotoxicity, whereas the cytotoxicity of ApoE4 is more than 80% when it is administered to cells at 1 μM. We wanted to test whether in vitro treatments of ApoE4 combined with the non‐toxic human chimeric 7C11 (7C11.chIgG1, Figure 4B) antibody would mimic the rescue in cytotoxicity observed when cells were treated with ApoE4Ch. To evaluate this, we used 1 μM ApoE as a reference for maximum cytotoxicity and compared the effect of 7C11.chIgG1 once incubated for 24 h with 1 μM ApoE4. As summarized in Figure S6, we tested different concentrations of 7C11. Overall, our data suggest that 6.7 nM 7C11.chIgG1 effectively reduces ApoE4‐derived cytotoxicity (Figure 4C). To validate our assay, we compared the effect of heparin treatment to that of 7C11.chIgG1. While both 2.1 nM 7C11 and 2.6 nM heparin significantly reduced ApoE4‐induced cytotoxicity as compared to 1 μM ApoE4 (ApoE4 + 7C11, *p* = .0097; ApoE4 + Heparin, *p* = .0295, one‐way ANOVA), we observed no statistical difference between these two treatment conditions (Figure 4C). Further, we confirmed that the newly designed antibody effectively recognized both mammalian‐ and *E. coli*‐produced ApoE (Figure S7) and that 7C11.mAb inhibited ApoE‐HSPG binding by testing the competition between 7C11 and heparin in a far‐western assay (Figure 4D) without significantly affecting ApoE‐VLDLr binding (Figure S8). In this far‐western assay, heparin was allowed to bind to ApoE in the membrane before blotting with the antibody. Our data confirmed that heparin effectively reduced 7C11 binding to both ApoE3 and ApoE4 without directly interfering with ApoE‐VLDLr binding (Figure 4D and Figure S8).

ApoE is a known ligand of HSPGs, which are known to interact with tau under specific pro‐aggregating conditions.^32^ Intriguingly, the protected *PSEN1* E280A carrier with *APOECh* homozygosity had high amyloid levels in her brain and very low tau, strongly suggesting a correlation between the loss of ApoE binding to HSPGs, reduced tau burden, and delayed onset of dementia in the presence of the *APOE3Ch* variant.^13^ Conversely, we and others have shown that the pathological ApoE4 has a stronger binding affinity to HSPGs, as modeled by heparin‐affinity chromatography.^13^, ^16^ Importantly, we recently showed that the link between reduced tau phosphorylation and ApoE Christchurch was direct using genetic analyses in induced pluripotent stem cell‐derived cerebral organoids.^33^ ApoE3Ch was linked to reduced tau phosphorylation in cerebral organoids with and without the *PSEN1* E280A mutation.

We reasoned that there was value in generating a rapid model to assess preclinical efficacy in vivo that required relatively small amounts of antibodies compared to systemic administration. Such a model may help in the assessment of biologics, which are expensive to produce. We generated a novel model that allowed for the rapid evaluation of the pre‐clinical efficacy of our antibodies on tauopathy using the mouse retina as a model (Figure 4E‐G). Even though our model has the limitation of not assessing changes in the brain, of note, one of the first in vivo demonstrations of Aβ toxicity published in *Nature* in 1998 was conducted via intravitreal injection of aggregated Aβ in the eye cavity of a rat, which triggered significant apoptosis in retinal cells.^34^ As noted in that classic paper, the retina is an integral part of the central nervous system, its structure is well organized, and because the eye is a closed system, small amounts of injected biologics remain in the vitreous body for extended periods of time, allowing for the assessment of efficacy.^35^, ^36^, ^37^, ^38^, ^39^ We designed our approach to specifically model human ApoE‐associated tau pathology using the mouse retina of B6;C3‐Tg(Prnp‐MAPT*P301S)PS19Vle/J (referred as P301S Tg) mice because of the robust protection to tauopathy in the Christchurch homozygote case. We would not expect our antibody to detect mouse ApoE in the P301S MAPT mouse model. This impossibility justifies our administration of human ApoE to trigger pathology in the eye.

This model shows definitive human ApoE‐dependent augmentation of tau phosphorylation: at 41 days of age this transgenic mouse has no detectable tau phosphorylation using the AT8 antibody in the retina, but increased signal is triggered by ApoE3 within 3 days following intravitreal injection. A pictographic representation of the experimental design is reported in Figure 4E.

As summarized in Figure 4E, we injected intravitreally P301S Tg mice with either ApoE3 (0.8 μg/mL) or ApoE3 and 7C11.mAb (950 μg/mL) and tested changes in tau hyperphosphorylation (AT8‐Tau) 3 days following 2 μL injection via immunofluorescence of flat mount retinas. Remarkably, compared to the vehicle, intravitreal injections of ApoE3 resulted in a significant accumulation of paired helical filaments of tau, thereby confirming that ApoE exacerbates tau pathology in the retina. This phenotype was rescued by injecting the anti‐ApoE antibody 7C11.mAb (Figure 4F). This finding suggested that blocking interactions between ApoE and HSPGs at the R136 site reduced ApoE3‐driven tauopathy in vivo.

Altogether, these experiments represent a critical experimental demonstration of the protective effects of mimicking the loss in HSPG binding of the Christchurch variant on ApoE‐mediated tauopathy, a finding consistent with our observations in the human case report.

### 3.6 Systemic delivery of 7C11 antibody is distributed in the brain and reduces tau phosphorylation

**FIGURE 5:**
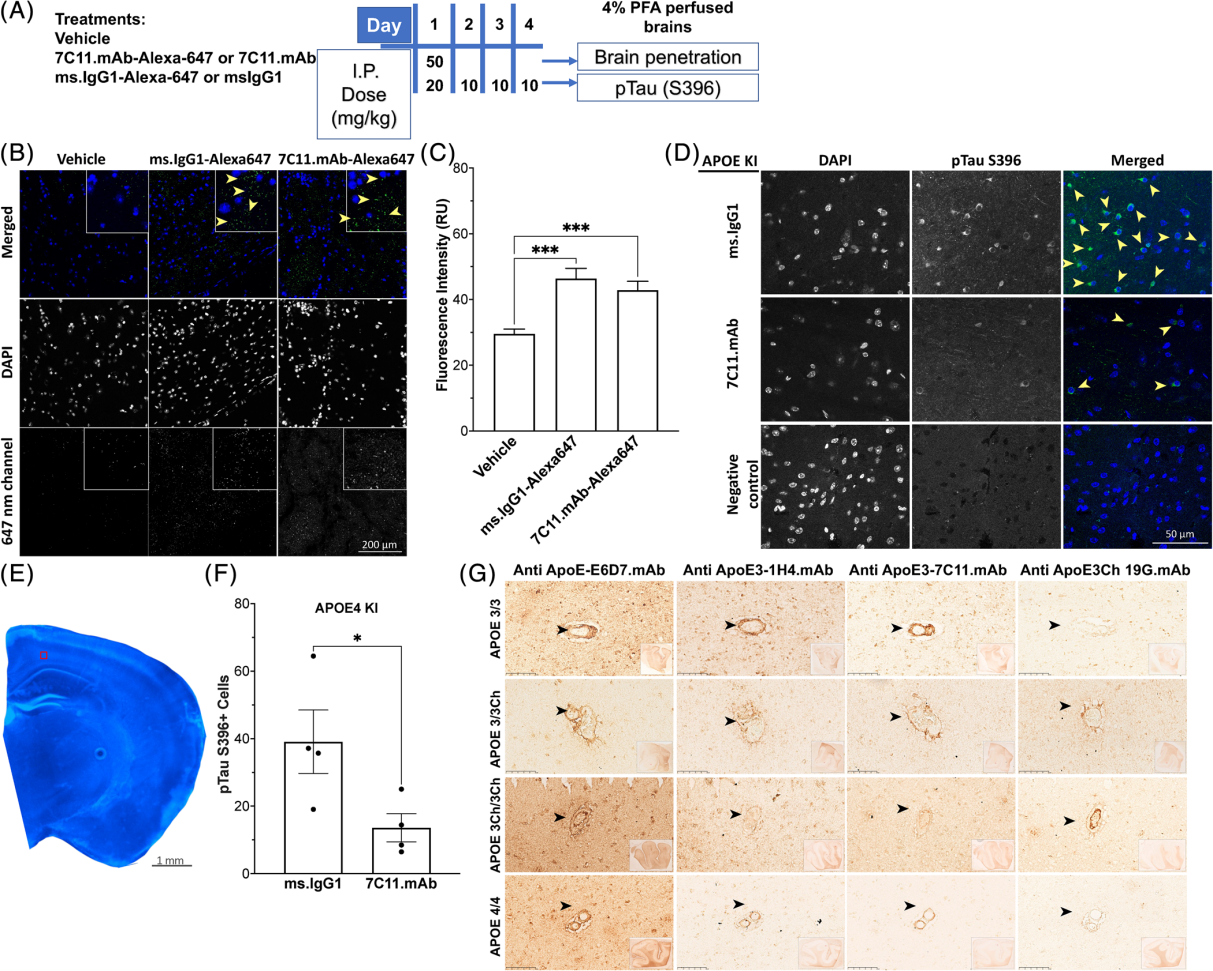
In vivo and human neuropathology characterization of new anti‐ApoE‐HSPG antibodies. (A) Pictographic representation of experimental design of intraperitoneal (ip) injections of WT and *APOE4* KI mice to determine brain penetration of Alexa‐647‐labeled antibodies (day 1, 50 mg/kg) or WT and *APOE4* KI mice to determine changes in pTau levels (days 1 to 4). (B) Representative staining of hippocampal regions of *APOE4* KI mice ip injected with either vehicle (PBS), ms.IgG‐Alexa647, or ms.7C11‐Alexa647 for 24 h, 50 mg/kg. Nuclei are stained with DAPI in blue. Scale bar = 200 μm. (C) Image analysis of injected brains shown in panel (B) was performed by measuring fluorescence intensity (FI) on 647‐nm channel. Higher FI of both ms.IgG1‐Alexa647 (*p* < .001, one‐way ANOVA) and 7C11.mAb‐Alexa647 (*p* < .001, one‐way ANOVA) injected groups was observed compared to vehicle. (D) Representative images of DAPI and pTau (S396) staining in APOE4 KI mice injected ip with either ms.IgG1 or 7C11.mAb, compared to negative control (secondary only antibody). Scale bar = 50 μm. (E) Representative coronal section stained with DAPI showing with a red square the anatomical region selected for the analysis presented in panel (F). Scale bar = 1 mm. (F) *APOE4* KI mice treated with 7C11 antibody showed a reduced number of pTau (S396)‐positive cells (*p* = .0286, one‐tailed Mann‐Whitney test). (B, D, E) Nuclei are stained with DAPI in blue. (G) Representative immunohistochemistry staining of ApoE levels on paraffin‐embedded temporal cortex specimens from PSEN1 E280A carriers with different *APOE* genotypes: *APOEε3/ε3* (first row), *APOE*ε3/ε3Ch (second row), *APOE*ε3Ch/ε3Ch (third row), *APOE*ε4/ε4 (last row). ApoE staining performed (from left to right) with anti‐ApoE‐E6D7, 1H4.mAb, 7C11.mAb, and 19G.mAb. Comparison of different staining patterns suggesting that, unlike the anti‐ApoE‐E6D7, which homogeneously stains ApoE variants throughout the tissue, 1H4.mAb preferentially stains ApoE3 and ApoE4 located either in the neuronal tissue or surrounding the vessels; 7C11.mAb preferentially detects ApoE3 and ApoE4 localized in deposits and surrounding the vessels, and 19G.mAb selectively stains ApoE3Ch. For each specimen analyzed, the region of the cerebral cortex used for the staining is reported in the bottom left boxes. Scale bar = 100 μm. Regions of interest in panels (B), (D), (G) are pointed with triangles.

To determine whether the 7C11 antibody could cross the blood‐brain barrier after systemic administration, we labeled 7C11.mAb antibody using Alexa‐647 a day prior to intraperitoneally injecting 50 mg/kg of either vehicle (PBS), Alexa‐647‐labeled mouse IgG1 isotype control, or Alexa‐labeled 7C11.mAb in 16‐month‐old *APOE4* knock‐in (KI) male mice (see Figure 5A for the experimental design). Twenty‐four hours after injection, we determined the biodistribution in the sagittal sections, including hippocampal regions, as the hippocampus is one of the regions mostly affected by AD^40^ and *APOE4* is the major risk factor for sporadic AD.^41^ As summarized in Figure 5B,C, both isotype control and 7C11.mAb were significantly distributed in the brain (vehicle vs ms.IgG1, *p* < .001, n1 = 30, n2 = 29, q = 6.64, DF = 86; vehicle vs 7C11.mAb, *p* < .001, one‐way ANOVA, n1 = 30, n2 = 30, q = 5.28, DF = 86), thereby validating the ability of our newly designed antibody to cross the blood‐brain barrier. We further tested whether ip injections of 7C11.mAb antibodies were able to reduce ApoE4‐induced tau hyperphosphorylation. Increased tau phosphorylation has been reported as a feature of ApoE4‐expressing mice^42^ (Figure S9). We reasoned that tau phosphorylation was a dynamic process, and as such, daily treatments for a short period of time may allow for observable differences of a biomarker. Specifically, we injected *APOE4* KI male and female mice for 4 days with either msIgG1 (*n* = 4, *APOE4* KI) or 7C11.mAb (*n* = 4, *APOE4* KI) with 20 mg/kg (day 1, to saturate depots) or 10 mg/kg (days 2 to 4) and quantified changes in pTau (S396) levels in the brain of these injected mice (Figure 5D, E). Our data show that levels in pTauS396 in *APOE4* KI mice are significantly reduced in the mice injected with 7C11.mAb as compared to IgG1‐injected mice (Figure 5E, *p* = .0286, one‐tailed Mann‐Whitney test). Overall, our data strongly support the 7C11 antibody as a promising candidate for future therapies targeting ApoE‐induced tau pathology.

### 3.7 Novel anti‐ApoE antibodies bind differentially to human ApoE variants in *post mortem* human tissue

We used the novel anti‐ApoE antibodies to stain temporal gyrus sections of autosomal dominant AD (ADAD) *PSEN1* E280A female carriers with either *APOE3*, *APOEε3/ε3Ch*, *APOEε3Ch/ε3Ch*, or *APOEε4/ε4* genetic variants (refer to Table S3 for basic demographic details). As positive control, we used the anti‐ApoE antibody clone E6D7 and compared staining obtained with 1H4.mAb, 7C11.mAb, and 19G.mAb antibodies. The observations described below are not quantifiable because we only had limited tissue from a small number of informative cases. We found that clone E6D7 stained ApoE homogeneously in human tissue from cases independently of the allelic variants present in specific cases including ApoeE3, ApoE4, and ApoE3Ch. In contrast, clone 1H4.mAb preferentially stained brain tissue from cases with ApoE3 and ApoE4 compared to tissue from the Christchurch homozygote case. Signal was focused on proteinaceous deposits with diffuse distribution throughout the neuronal tissue and in the vessels. Interestingly, we observed more localized and apparently specific staining when we used 7C11.mAb. As expected, 7C11.mAb showed a signal that was strong in tissue from ApoE3 and ApoE4 carriers but less robust in tissue from the Christchurch homozygote case. Conversely, clone 19G.mAb selectively stained ApoE3Ch with vascular localization as confirmed by the positive stain only in the specimens obtained from the *PSEN1*E280A carriers with *APOEε3/ε3Ch* and *APOEε3Ch/ε3Ch* genotypes.

## 4 DISCUSSION

Prior to our work with *APOE* Christchurch, it was unknown that a reduction of ApoE‐HSPG interactions could potentially result in a profound phenotype of resistance to AD in humans for almost three decades. Others have developed antibodies specific for ApoE by targeting different epitopes of ApoE away from the HSPG‐binding domain.^43^, ^44^ This alternative approach seeks depletion of ApoE, whereas our mAb seeks competitive inhibition of the interaction between ApoE and GAGs at the ApoE Christchurch site, as confirmed via affinity chromatography and far‐western blotting assays (Figures 3A,B and 4D).

As we have learned from the amyloid‐targeting efforts, it is impossible to know ahead of time which antibodies targeting ApoE will be safer and more effective as therapeutics. Multiple ApoE‐targeting approaches should be pursued in parallel to increase our chances of developing a product that will help patients in the end. Based on the reduction of ApoE‐induced tau phosphorylation using an in vivo model of intravitreal injection in a mouse model of tauopathy (Figure 4) and with ip injection in a humanized *APOE4* KI mouse model (Figure 5), our approach holds the promise of clinical impact. Our approach is inspired by a case‐based observation of a three‐decade delay in the age of onset of cognitive decline for one of the most aggressive forms of AD.

In this work, we described the design and validation of novel antibodies specifically targeting the region of the HSPG‐binding domain of ApoE and ApoE3Ch (Figure 1). Using binding assays, we confirmed that the library of antibodies differentially targeted the different ApoE variants (Figure 2), thereby offering a novel platform for targeted therapeutics. Using in vitro assays, we determined the effective dose of 7C11 antibody that reduced ApoE4‐HSPG binding, which is known to be upstream of ApoE‐induced mitochondrial dysfunction and ER stress,^45^, ^46^ which ultimately led to the observed cytotoxicity (Figure 4). Overall, our findings strongly suggest that the interaction between 7C11 and ApoE variants might be dependent on conformations that ApoE can assume in solution (eg, oligomeric), which are less favored when the proteins are bound to the HS1K chip. Such dynamic conformations are certainly related to the amino acid difference in ApoE4 (Cys158 → Arg158) and ApoE2 (Cys112 → Arg112) as compared to ApoE3. These conformations might therefore affect the three‐dimensional structure of the proteins and the availability of the heparin binding domain, resulting in the different interaction between ApoE variants and 7C11 antibody.

Oligomeric/aggregated ApoE has been shown to have toxic properties in vitro and in vivo.^47^ Since ApoE4 is the strongest genetic risk factor for sporadic AD,^48^ the observed increased binding affinity between 7C11.mAb and ApoE4 in solution may be therapeutically relevant. Many AD patients carry *APOE3*, so the high affinity of our antibodies for ApoE3 is also clinically relevant.

Additional studies that involve a longer treatment duration and other animal models, will be useful to confirm preclinical efficacy. Specifically, the effects of the 7C11 antibody in the presence of amyloid and advanced tauopathy (we only examined tau phosphorylation) remain to be examined.

We conclude that we have successfully designed a library of anti‐ApoE antibodies targeting ApoE with different degrees of sensitivity and selectivity. We anticipate that the therapeutic potential of the antibody against *ApoE3Ch* will be limited; nevertheless, ApoECh‐specific antibodies can be used for research purposes that could differentially detect both the protective (ApoE3Ch) and detrimental (ApoE4) variants in vivo and in neuropathology specimens. Further, given the strong affinity of the top candidate 7C11.mAb for ApoE3 and ApoE4 (Figure 5), our study can contribute to future disease‐modifying therapies relevant for AD and other neurodegenerative conditions via reduction in tau pathology.

## AUTHOR CONTRIBUTIONS

C. Marino and J.F. Arboleda‐Velasquez designed the experimental approaches and drafted the manuscript. C. Marino and P. Perez‐Corredor conducted most of the experiments and analyses. M. O'Hare, L. Gonzalez‐Buendia, S. Delgado‐Tirado, T. H. Doan contributed and H. Gordon contributed to the in vivo experiments. A. Heuer and N. Chmielewska contributed to the chromatography experiments, A. Chandrahas contributed to the in silico analyses, C. Ortiz‐Cordero contributed to the in vitro experiments. S. Arevalo‐Alquichire and T. Vanderleest contributed to the statistical analyses. A. Villegas, D. Sepulveda‐Falla, F. Lopera and P. Perez‐Corredor contributed to collecting and analyzing the *post mortem* tissue; Y. T. Quiroz, R. Mahley, L.A. Kim, R. A. Obar, D. Sepulveda‐Falla and Y. Huang contributed by providing critical inputs to finalize the manuscript.

## CONFLICT OF INTEREST STATEMENT

Drs Y. Quiroz, F. Lopera and J. Arboleda‐Velasquez are inventors on a patent filed by Mass General Brigham to leverage therapeutics inspired by the APOE Christchurch findings. Dr Y. Quiroz received grants from the National Institute on Aging, the Alzheimer's Association and Massachusetts General Hospital ECOR. Dr Y. Quiroz is and Editorial Board Member of this journal but was not involved in the peer‐review process nor had access to any information regarding its peer‐review and serves as a consultant for Biogen. Drs. J. Arboleda‐Velasquez and L. A. Kim are co‐founders of Epoch Biotech, an LLC developing resilient case‐inspired therapeutics. F. Lopera received consulting fees from Biogen and Tecnoquimicas. All other authors have no conflicts to disclose. Author disclosures are available in the supporting information.

## CONSENT STATEMENT

Informed consent from human subjects was obtained for all tissue received.

## Supporting information

Supporting Information

Supporting Information
