# Supplementary material for: APOE Christchurch‐mimetic therapeutic antibody reduces APOE‐mediated toxicity and tau phosphorylation
Source: Alzheimers Dement. 2023 Oct 4;20(2):819–36. doi: 10.1002/alz.13436 (PMC10916992; doi:10.1002/alz.13436)
Supplement: Supplementary file 1 — Supporting Information [file ALZ-20-819-s001.docx]

**Supplementary Appendix**

*Marino C. et al., 2023.*

**Figure S1**. **Statistical comparisons of 7C11.mAb and 1H4.mAb binding to ApoE3.** Analysis or the Area Under Curve (AUC) calculated for the binding profiles presented in figures 1E, F showing that 7C11.mAb binding to ApoE3 full-length is significantly higher than 1H4.mAb (purple bars, p = 0.0177, one-way ANOVA followed by Tukey test for multiple comparisons). Both antibodies showed a significantly higher binding to the ApoE3 peptide as compared to ApoE3 FL (green bars, 7C11.mAb-ApoE3 Peptide vs. 7C11.mAb-ApoE3 FL p < 0.00001, q = 20.51; 1H4.mAb-ApoE3 Peptide vs. 1H4.mAb-ApoE3 FL, p < 0.0004, q = 28.13, n1 = n2 = 21, DF 80).

**Figure S2**. **ELISA screening of anti-ApoE3Ch antibodies binding to ApoE**. (A-B) Binding analyses of supernatant of hybridoma cells immunized with ApoE3Ch expressing 19G.Ab or 25F.Ab clones tested using ApoE3 and ApoE3Ch, and both HSPG-binding region peptides (referred as peptide) (A) and full length (FL) proteins (B). Data confirmed the specificity of these antibodies for the ApoE3Ch peptide, but less specific binding for FL ApoE3Ch as compared to ApoE3. Data presented as averaged binding % as a function of logarithm of dilution factor ± s. e. m. of n = 3 repeated measures. (C-D) ELISA binding profiles of purified monoclonal antibodies 19G.mAb and 25F.mAb tested for their binding to either FL protein (ApoE3 in purple and ApoE3Ch in magenta) or peptide of both ApoE3 (in green) and ApoE3Ch (in cyan). Data shows the differential binding for the clones for either full length or small peptides of ApoE3 and ApoE3Ch.

******

**Figure S3**. **ELISA validation of the anti-ApoE3Ch antibodies with ApoE variants and binding analysis via BLI**. (A, B) ELISA binding profiles of 19G.Ab (A), 25F.mAb (B) tested with ApoE2, ApoE3, ApoE4 and ApoE3Ch validating the specificity of both 19G.mAb and 25F.mAb for ApoE3Ch. (C) ELISA binding profile of mouse IgG1 control tested with ApoE variants as negative control for the ELISA assays presented in Figure 2 and S2. (D, F) Binding analysis obtained via titration of the anti ApoE3-HSPG antibody 7C11.mAb tested with ApoE variants (ApoE2, D; ApoE3, E; ApoE4, F) coated on HS1K chip via BLI. KD reported inside the graphs (G) BLI profiles for the binding between 19G.mAb and ApoE2 obtained using HS1K chip.

***Table*** ***S1****. Analysis of the half maximal concentration of the anti-ApoE antibodies tested for the binding to ApoE variants.*

| ApoE Variant → | **ApoE2** | | | | |
| --- | --- | --- | --- | --- | --- |
| Anti ApoE clones ↓ | LogEC50 | | EC50 (nM) | | |
|  | Value | SEM | Value |  | |
| **7C11** | -1.055 | 0.017 | 0.088 |  | |
| **1H4** | -1.068 | 0.010 | 0.086 |  | |
| **D6E10** | -1.089 | 0.141 | 0.082 |  | |
| **19G** | 4.507 | 0.284 | 32158.113 |  | |
| **Anti-His tag** | -0.771 | 0.021 | 0.169 |  | |
| ApoE Variant: | **ApoE3** | | | | |
|  | LogEC50 | | EC50 (nM) | | |
| Anti apoE clones: | Value | SEM | Value | |  |
| **7C11** | -0.679 | 0.059 | 0.209 | |  |
| **1H4** | -0.796 | 0.035 | 0.160 | |  |
| **D6E10** | -1.130 | 0.161 | 0.074 | |  |
| **19G** | 4.998 | 0.919 | 99632.010 | |  |
| **Anti-His tag** | -0.762 | 0.014 | 0.173 | |  |
| ApoE Variant: | **ApoE4** | | | | |
|  | LogEC50 | | EC50 (nM) | | |
| Anti poE clones: | Value | SEM | Value | |  |
| **7C11** | -0.810 | 0.027 | 0.155 | |  |
| **1H4** | -0.961 | 0.022 | 0.109 | |  |
| **D6E10** | -1.181 | 0.132 | 0.066 | |  |
| **19G** | 3.569 | 0.062 | 3707.745 | |  |
| **Anti-His tag** | -0.739 | 0.032 | 0.183 | |  |
| ApoE Variant: | **ApoE3Ch** | | | | |
|  | LogEC50 | | EC50 (nM) | | |
| Anti apoE clones: | Value | SEM | Value | |  |
| **7C11** | 3.528 | 0.331 | 3376.293 | |  |
| **1H4** | 3.162 | 0.221 | 1452.824 | |  |
| **D6E10** | -1.076 | 0.159 | 0.084 | |  |
| **19G** | 1.633 | 0.117 | 42.908 | |  |
| **Anti-His tag** | -0.758 | 0.024 | 0.175 | |  |

******

***Figure S4. ELISA validation of the anti-ApoE3Ch antibodies with mouse ApoE.*** *ELISA binding profiles of 1H4.mAb (magenta), 7C11.mAb (cyan), 25F.mAb (purple), 19G.Ab (light purple), tested with mouse ApoE (ApoEms) confirming that only the anti His-tag antibody (His.mAb) binds to the ApoEms, thus confirming the specificity of the newly designed antibodies for human ApoE variants. Data presented as binding percentage over Logarithmic nanomolar concentration (Log C, nM).*

***Supplementary Figure S5. Affinity chromatography analysis of ApoE3 and ApoE4 in the presence of 7C11 antibody.*** *(A, B)* *Representative chromatograms of 7C11.mAb 0.6 µM (A, cyan), ApoE3 1.47 µM and ApoE3 1.47 µM (A, magenta) incubated with 7C11.mAb 0.6 µM (A, blue) or 7C11.mAb 0.6 µM (B, cyan), ApoE4 1.47 µM (B, purple) and ApoE4 1.47 µM incubated with 7C11.mAb 0.6 µM (B, blue). Data is showing that 0.6 µM concentration of 7C11.mAb has a strong inhibitory effect of the heparin binding of ApoE3 and ApoE4 as confirmed by the shift of p_max_ at lower retention times and reduction of the peak intensity as compared to ApoE alone. All chromatograms are expressed as normalized intensities to the maximum intensity of emission over time in minutes (min.) and are representative of three independent measurements. Chromatographic elution of vehicle has been used for baseline correction.* *Salt gradient percentage of 0.8 M NaCl is represented by the dotted line.*

***Supplementary Table 2****. Crystallographic analysis of the Fab of the anti-ApoE 7C11 antibody*

| **Summary of data collection and reﬁnement statistics** | |
| --- | --- |
| Target protein | 7C11 IgG Fab |
| Wavelength | 0.9792 |
| Space group | P 21 21 2 |
| a,b,c (Å) | 92.700   62.270   82.140 |
| α,β,γ (°) | 90 90 90 |
| Resolution (Å) | 26.26- 1.70 (1.76 - 1.70) |
| No. of unique reflections | 699695 (71798) |
| Completeness (%) | 99.94 (99.99) |
| <I>/δ(I) | 27.41 (5.54) |
| Redundancy | 13.2 (13.8) |
| R_merge_ | 0.053 (0.726) |
| R_p.i.m_ | 0.015 (0.203) |
| R_work_ /R_free_ | 0.185/ 0.205 |
| No. atoms | 3756 |
| Protein | 3310 |
| Ligand | 14 |
| Water | 432 |
| B-factors | 33.03 |
| Protein | 31.96 |
| Ligand | 48.36 |
| Water | 40.73 |
| **X-ray Diffraction data quality analysis** | |
| Ramachandran |  |
| Favored (%) | 97.86 |
| Allowed (%) | 2.14 |
| Outlier (%) | 0 |
|  |  |
| R.m.s. deviations |  |
| Bond lengths (Å) | 0.008 |
| Bond angles (°) | 1.11 |

***Figure S6. Cytotoxicity profile of serial dilutions of 7C11 in the presence of 1μM ApoE4.*** *LDH assay of neuroblastoma cells (SH-SY5Y) treated 24 h with either increasing doses of 7C11.chIgG1 (7C11), magenta curve) or ApoE4 1 μM co-administered with increasing nanomolar (nM) concentrations of 7C11.chIgG1 antibody (cyan curve, 7C11 + 1 μM ApoE4). Data is expressed as percentage of cytotoxicity using as controls untreated cells and fully lysates cells and normalized to ApoE4 1 μM cytotoxicity expressed as 100%.*

***Figure S7. 7C11 antibody binds to ApoE variants produced in mammalian and bacteria ApoE variants.*** *Representative western blotting of vehicle (PBS), recombinant ApoE variants produced in E. coli (non-glycosylated, in blue) and in mammalian cells (HEK derived, glycosylated in green). In the top blot, proteins were probed with 7C11.mAb antibodies; in the bottom blots, proteins were probed with anti-ApoE antibody (bottom blots).*

***Figure S8. In silico and ELISA analysis of the effect of 7C11.mAn on ApoE-VLDLr binding.*** *(A)*

*Representative in silico polar contact analysis of ApoE3-VLDLr structures. Amino acids required for polar contacts are highlighted in yellow in the sequences below the structure. The region of the Christchurch mutation is highlighted in bold. (B) Binding analysis of ApoE-VLDLr in the presence (blue profiles) or the absence of 7C11.mAb (red) is used as a competitive antagonist at the molar ratio highlighted in cyan above the x-axis. Data were normalized to the maximum 450 nm absorbance, and it is expressed as a binding percentage over the logarithmic concentration of ApoE3 (LogC, nM). Data sets were fitted to a four parameters sigmoidal model, and Akaike’s Information Criterion assessed the statistical difference between the models. One curve with the parameters enlisted in the plot (dotted black curve) was able to describe both data sets. The area under the curve (AUC) was calculated for eight replicates of each condition, and the statistical difference was evaluated with an unpaired t-test. There was no significant difference between the AUC (p = 0.3174).*

***Figure S9. Phosphorylated tau levels in wild type and APOE4 KI mice.*** Representative images of pTau (S396) staining in wild type (WT) and APOE4 KI mice. left panel reports nuclear DAPI staining, middle panel pTauS396 staining and right panel the merged channels. Yellow triangles highlight Tau-positive cells. Yellow triangles highlighting some of the pTau-positive cells. Scale bar = 200 µm.

*Table S3.* ***Demographic information of PSEN1 E280A carriers used for histological analysis presented in Figure 5.*** *APOE background, sex, age of dementia onset (AoO), age of death (AoD), postmortem index (PMI) and time stored (TS).*

| **APOE** | **PSEN1** | **Sex** | **AoO** | **AoD** | **PMI** | **TS** |
| --- | --- | --- | --- | --- | --- | --- |
| ε3ε3 | E280A | F | 50 | 59 | 4h, 05 min. | 7 years, 2 months |
| ε4ε4 | E280A | F | 35 | 48 | 2h, 45 min. | 16 years, 3 months |
| ε3ε3Ch | E280A | F | 50 | 55 | 1h, 45 min. | 3 years, 11 months |
| ε3chε3Ch | E280A | F | 75 | 76 | 3h, 20 min. | 2 years, 3 months |
